# A machine learning approach to determine the influence of specific health conditions on self-rated health across education groups

**DOI:** 10.1186/s12889-023-15053-8

**Published:** 2023-01-18

**Authors:** Jordi Gumà-Lao, Bruno Arpino

**Affiliations:** 1 Department of Sociology, Umeå University, Vindarnas torg 1 Beteendevetarhuset, Umeå, 901 87 Sweden; 2 Department of Statistics, Computer Science, Applications, University of Florence, Viale Morgagni, 59, Firenze, 50134 Italy

**Keywords:** Self-rated health, Health conditions, Education, Machine learning, SHARE survey

## Abstract

**Background:**

Self-rated health, a subjective health outcome that summarizes an individual’s health conditions in one indicator, is widely used in population health studies. However, despite its demonstrated ability as a predictor of mortality, we still do not full understand the relative importance of the specific health conditions that lead respondents to answer the way they do when asked to rate their overall health. Here, education, because of its ability to identify different social strata, can be an important factor in this self-rating process.

The aim of this article is to explore possible differences in association pattern between self-rated health and functional health conditions (IADLs, ADLs), chronic diseases, and mental health (depression) among European women and men between the ages of 65 and 79 according to educational attainment (low, medium, and high).

**Methods:**

Classification trees (J48 algorithm), an established machine learning technique that has only recently started to be used in social sciences, are used to predict self-rated health outcomes. The data about the aforementioned health conditions among European women and men aged between 65 and 79 comes from the sixth wave of the Survey of Health, Ageing and Retirement in Europe (SHARE) (*n* = 27,230).

**Results:**

It is confirmed the high ability to predict respondents’ self-rated health by their reports related to their chronic diseases, IADLs, ADLs, and depression. However, in the case of women, these patterns are much more heterogeneous when the level of educational attainment is considered, whereas among men the pattern remains largely the same.

**Conclusions:**

The same response to the self-rated health question may, in the case of women, represent different health profiles in terms of the health conditions that define it. As such, gendered health inequalities defined by education appear to be evident even in the process of evaluating one’s own health status.

## Background

Self-rated health as an indicator of an individual’s health status has received considerable attention because of its demonstrated ability as a predictor of mortality [1, 2]. Although there is a large number of studies on the possible influence of health indicators on SRH, systematic approaches to examine the relative importance of various indicators at the same time are rather scarce. This implies that we have a high level of knowledge about the specific effects of different health determinants, but we still have a limited understanding about their relative importance when these factors are analyzed together [3]. This reduces the understanding of what is measured by self-rated health. Indeed, because of its subjective nature, and the fact that it summarizes all the health conditions of an individual in just one indicator, self-rated health remains something of a black box.

A number of studies have sought to shed light on the nature of the relationship between this indicator and specific aspects of health, including chronic conditions [4], functional health [5], and depression [6]. Notwithstanding, in a recent study of the structure of self-rated health among the nonagenarian population of Finland, Lisko et al. [7] did find a direct association between certain health conditions – including fatigue, depression, mobility problems, dizziness, vision deficits, and heart disease – and poor self-rated health. Complementarily, a conjunction between functional capacities, chronic and mental illnesses also proved to associate with answers about self-rated health [8].

Despite this evidence pointing to the influence of certain objective health conditions, self-rated health cannot be fully understood without analyzing the subjective processes that lead people to respond the way they do when questioned about their overall health. Of note here is the contribution of Jylhä [9] who identifies three key factors influencing an individual’s perception: the individual’s health biography, the reference groups the individual takes into consideration when making an assessment (How do I compare healthwise in relation to my peers?), and the cultural conventions that can condition the individual’s response. In this research, we focus mainly on the second factor, the reference group based on their socioeconomic characteristics.

When comparing people with similar sociodemographic characteristics, there is probably a process of homogenization of the subjective conception of health within the same group. This process, known as social comparison, permits to understand the more optimistic assessment of health in general of older population, among whom the normalization of negative health situations has led to a more favorable evaluation of their own health [10]. Social comparison could be influenced by the same factors that act as social determinants of health, mainly among population within the same age group. For example, the higher prevalence of health problems among people with a disadvantaged sociodemographic profile may lead to the normalization of this situation, and therefore to a less negative subjective perspective. In this research, we focused on education as a factor that could potentially establish differences between individuals in how health determinants associate with self-reported health as a result of social comparison.

The aim of this article is to explore possible differences in association pattern between self-rated health and functional health conditions (IADLs, ADLs), chronic diseases, and mental health (depression) among European between the ages of 65 and 79 and whether this association varies according to educational attainment (low, medium, and high), separately for women and men. Education was chosen due to its ability to establish different levels of social stratification [11, 12]. Although population groups defined by their low level of education have been found to present greater disadvantages in terms of their self-rated health [13, 14], we are interested in knowing whether health conditions associate in the same way with the response on perceived health according to education.

To identify potential differences in association patterns between self-rated health and the selected health conditions, machine learning methods based on classification trees, namely random forests and the J48 algorithm (the updated version of the C4.5 algorithm), are applied to European individuals who participated in the sixth wave of the Survey of Health, Ageing and Retirement in Europe (SHARE) [15]. The J48 tree algorithm was specifically chosen as it provides a graphical representation of the relationship between variables predicting self-rated health. This representation, in turn, provides information about the degree of importance of each of these variables (the order of importance is established within the tree, the higher up the variable appears, the greater the importance), and the interaction between them, for making the final prediction. This is a non-parametric method, which allows great flexibility in determining the contribution of each variable to predict the values of the dependent variable (i.e., self-rated health in this specific case). This higher flexibility permits to identify different and complex patterns in the relationships between variables within each branch of the classification tree. Therefore, and differently from parametric methods, this approach allows to automatically capturing interactions and non-linearities in the association between the predictors and the outcome. All calculations were made separately by sex in order to account for gender differences on both health profiles and education.

## Methods

The data analyzed herein are taken from the sixth edition of the Survey of Health, Ageing and Retirement in Europe (SHARE), conducted in 2015. SHARE is a panel survey that is representative of the non-institutionalized population in Europe aged 50 and over. It gathers information about multiple aspects of this population, including demographics, work, family, health, housing, etc. [15]. Although two more waves of the survey have subsequently been conducted, the sixth is the most recent edition of the survey in which all questions included in the general panel survey questionnaire were comprehensively asked to all survey participants (note, the seventh wave was conducted to collect biographic information from respondents that did not participate in the third wave) and in which the respondents were not influenced by the COVID-19 pandemic (note, we also excluded wave 8 that was interrupted because of the emergence of the COVID-19 pandemic).

The working sample comprised all persons aged between 65 and 79 years old residing in the 17 European countries (Israel was discarded from the analysis) participating in the sixth edition of SHARE who answered all the questions included in our analysis (*n* = 27,230 from an original sample of 28,790, 94.6% with complete information). This age range was set for two main reasons in order to reduce possible sources of bias in our results: first, a minimum age of 65 years was used to account for the known effect of retirement on health perception and self-assessment of health. Individuals’ perception is substantially alleviated by retirement [16]. Therefore, including both working-age and retired individuals could affect our results; second, the age boundary of 79 was fixed because this has been shown to be the age at which the relationship between mortality and self-perceived health begins to weaken [17]. This would be an indication of a separation in the trend of self-rated health and objective health conditions. Therefore, we aim to reduce the influence of other characteristics of the reference group such as age or employment status by selecting this specific age group.

The choice of the four health indicators chosen to explore their relationship with self-rated health is based on their previous proven relationship with this indicator. The presence of chronic diseases has shown to affect the self-rated health of women and men differently [4]. This is because women’s and men’s specific chronic diseases are different. This is the reason why this variable has been dichotomized into two categories (having or not having any chronic disease) since the selection of certain more specific chronic diseases could lead to selection biases in one of the two sexes. As for IADLs and ADLs, both have been shown to influence self-rated health [18]. ADL associates with higher level of dependency, so including both we take into account not only the fact of presenting a certain limitation but also the severity of this limitation [19]. Finally, depression has also proved to be related to self-rated health [6] and allows us to incorporate the mental health dimension into our analysis.

All four health outcomes were dichotomized so as to facilitate interpretation of the resulting classification trees:

- Chronic disease. The SHARE questionnaire includes questions about 21 diagnosed chronic diseases and health conditions (heart attack or any other heart problem; high blood pressure or hypertension; high blood cholesterol; stroke or cerebral vascular disease; diabetes; chronic lung disease; cancer or malignant tumor; stomach or duodenal ulcer, peptic ulcer; Parkinson disease; cataracts; hip fracture; other fractures; Alzheimer’s disease, dementia, organic brain syndrome, senility or any other serious memory impairment; other affective or emotional disorders; rheumatoid Arthritis; Osteoarthritis, or other rheumatism; and chronic kidney disease). Here, the final variable was dichotomized as no chronic disease and chronic disease (i.e., one or more diagnosed chronic diseases).
- Activities of daily living (ADL): Participants were asked whether, “because of physical, mental, emotional, or memory problems”, they had any difficulty doing these activities (again, excluding any difficulties expected to last less than three months): dressing (including putting on shoes and socks); eating (such as cutting up your food); using the toilet (including getting up and down); bathing and showering; getting in and out of bed; and walking across a room. The final variable was dichotomized as no limitations and limited (having difficulties to perform one or more of the activities).
- Instrumental activities of daily living (IADL). Participants were asked whether they had any difficulty doing each of the following everyday activities: doing work around the house or garden; leaving the house independently/accessing transportation; shopping for groceries; doing personal laundry; managing money; preparing a hot meal; taking medications; and making telephone calls. Individuals were required to exclude any difficulties expected to last less than three months. The final variable was dichotomized as no limitations and limited (having difficulties to perform one or more of the activities).
- Depression: This variable was measured using the EURO-D scale, developed and validated by the EURODEP Concerted Action Project [20]. EURO-D compiles binary information about 12 different symptoms of depressive moods: depression, pessimism, wishing death, guilt, sleep, interest, irritability, appetite, fatigue, concentration, enjoyment, and tearfulness [21]. The scale ranges from 0 to 12 with a score above 3 representing significant depression levels [22]. The final variable was dichotomized as not depressed (values of 3 or lower) and depressed (values above 3).
- Self-rated health: The variable was based on responses to the question ‘How is your health in general?’ (excellent; very good; good; fair; poor). Following common practice [23], the five possible answers were grouped into two categories: excellent, very good or good health (good health), and fair or poor health (poor health).
- Education: The original information was coded using the International Standard Classification of Education, that is, the ISCED 1997 scale, designed by UNESCO to facilitate cross-country comparisons. The original values were grouped as follows: low (corresponding to ISCED 0–2, lower secondary education or lower), medium (ISCED 3–4, higher secondary education), and high (ISCED 5–6, post-secondary education).

The classification algorithm selected was the J48 [24], an updated version of the algorithm C4.5 proposed by Quinlan [25]. This algorithm belongs to the group of classification trees [26] whose objective is to determine how different variables are hierarchically related to predict a certain outcome. The decision tree is built starting with the variable with the highest discriminatory capacity in the final classification. This first variable establishes the root node. Taking this variable, the decision tree can then be branched to show all possible routes, thus illustrating the interrelation between variables, and resulting in the prediction of one outcome or another. The order of the variables within each of the branches informs about the relative importance of each attribute for predicting the outcome until the algorithm manages to reach a prediction that can be considered reliable. In the case of decision trees defined by categorical variables – as is the case here – the relationships between the different nodes in the classification tree are established logically by answering the question as to whether a respondent presents a certain characteristic or not for each of the nodes.

The k-fold cross-validation procedure was used in order to avoid overfitting, i.e., when the noise in the training data has a relatively high influence on the learning process of the model and the final model is too specific for this training data [27]. Under k-fold cross-validation the data are randomly partitioned into k different subsets of approximately equal size. In the i^th^ fold of the cross-validation procedure, the i^th^ subset is used to test the performance of a model trained on the remaining k − 1 subsets. In that way, all the different folds contribute to the training and validation process in different steps. In this case, the number of folds was set at 10. Complementarily, overfitting was controlled by pruning the decision trees to avoid branches that might be too complex and specific to the subsample analyzed in each case [28].

The predictive capacity of the J48 algorithm was validated by comparing the accuracies (% of successes, defined as the number of coincidences between real and predicted outcomes) of each of the models obtained using this algorithm with the values obtained from random forest (RF). RF fits an ensemble of decision trees and combines the results of them [29], what has been shown to minimize possible classification errors since it does not focus on a single tree [30]. However, it is not possible to have a graphical representation with the relationships between the different variables to predict the final result with RF as being composed of a combination of different classification trees. Therefore, it is used here as a benchmark to identify the level of accuracy of the results based on the J48 algorithm.

All the algorithms and their respective measures of accuracy were implemented using the Waikato Environment for Knowledge Analysis (WEKA) [31] software version 3.8.5.

## Results

Table 1 displays the health profile of the sample analyzed according to sex and educational level. In general, the worse health profile of women compared to men is confirmed. In all five health indicators, women show worse values, regardless of their educational level. However, the smallest differences are observed when comparing women and men with a high level of education, except in the case of depression. In fact, regardless of educational level, the greatest difference by gender is observed in the case of depression, being in all cases greater than 10% units.

**Table 1:** Health profile of individuals aged between 65 and 79 years old according to gender and education

	Women			Men		
	Low	Medium	High	Low	Medium	High
**Self-rated health**						
Good	48.4%	58.8%	71.5%	53.7%	62.2%	72.6%
Poor	51.6%	41.2%	28.5%	46.3%	37.8%	27.4%
** Total**	**100%**	**100%**	**100%**	**100%**	**100%**	**100%**
**Chronic diseases**						
No chronic disease	12.4%	16.6%	20.8%	16.7%	18.4%	22.1%
Chronic disease	87.6%	83.4%	79.2%	83.3%	81.6%	77.9%
** Total**	**100%**	**100%**	**100%**	**100%**	**100%**	**100%**
**IADL**						
No limitations	75.8%	83.5%	87.5%	84.6%	88.9%	90.9%
Limited	24.2%	16.5%	12.5%	15.4%	11.1%	9.1%
** Total**	**100%**	**100%**	**100%**	**100%**	**100%**	**100%**
**ADL**						
No limitations	86.5%	90.0%	93.0%	89.1%	90.5%	93.5%
Limited	13.5%	10.0%	7.0%	10.9%	9.5%	6.5%
** Total**	**100%**	**100%**	**100%**	**100%**	**100%**	**100%**
**Depression**						
Not depressed	60.7%	70.5%	76.8%	77.8%	83.6%	86.2%
Depressed	39.3%	29.5%	23.2%	22.2%	16.4%	13.8%
** Total**	**100%**	**100%**	**100%**	**100%**	**100%**	**100%**
**N**	**6 934**	**5 227**	**2 642**	**4 642**	**4 755**	**3 030**
**% (according to gender)**	**46.8%**	**35.3%**	**17.8%**	**37.4%**	**38.3%**	**24.4%**

Data: 6th wave SHARE survey

As for the results by educational level, we observe in all the cases a clear health gradient according to education, with lower educated individuals showing worse health results. However, the magnitude of these differences is much greater in the case of women, again with higher differences in the case of depression, especially between low and medium educated population.

**Fig. 1:**
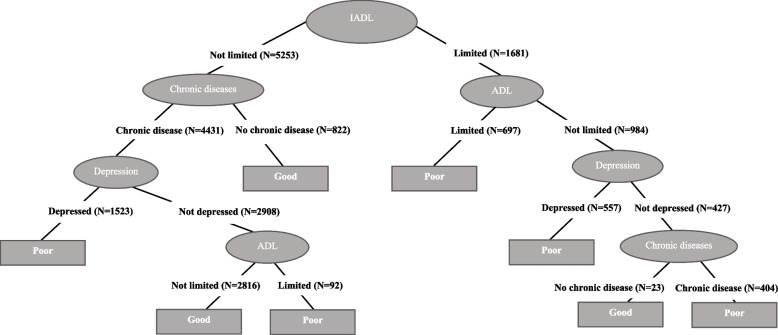
Decision tree for self-perceived health. Low educated women

**Fig. 2:**
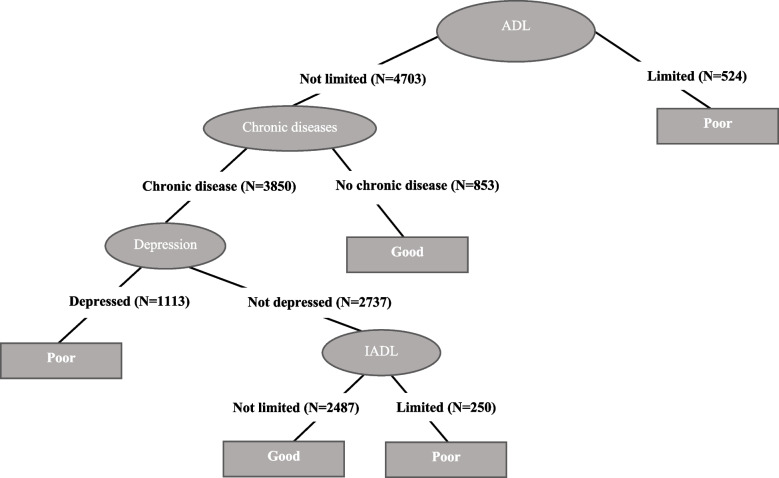
Decision tree for self-perceived health. Medium educated women

**Fig. 3:**
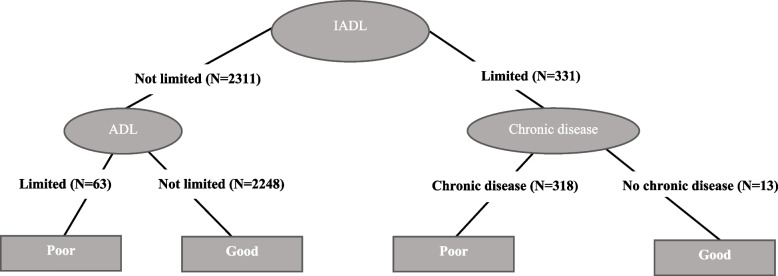
Decision tree for self-perceived health. High educated women

**Fig. 4:**
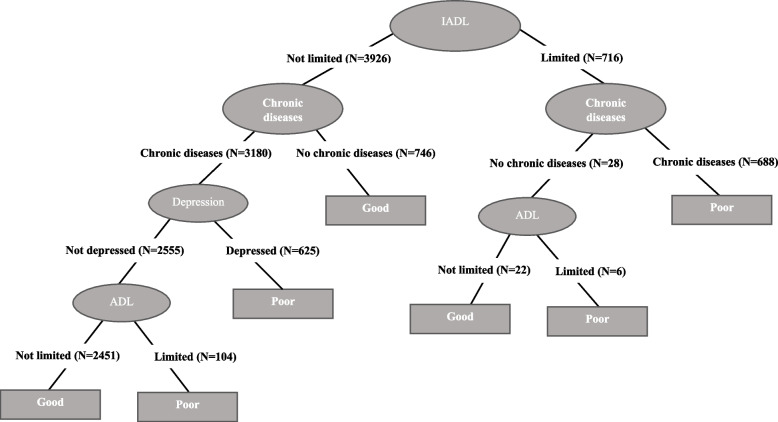
Decision tree for self-perceived health. Low educated men

**Fig. 5:**
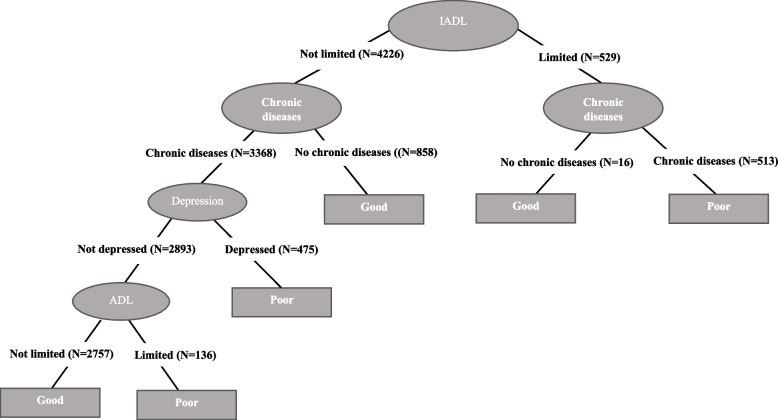
Decision tree for self-perceived health. Medium educated men

**Fig. 6:**
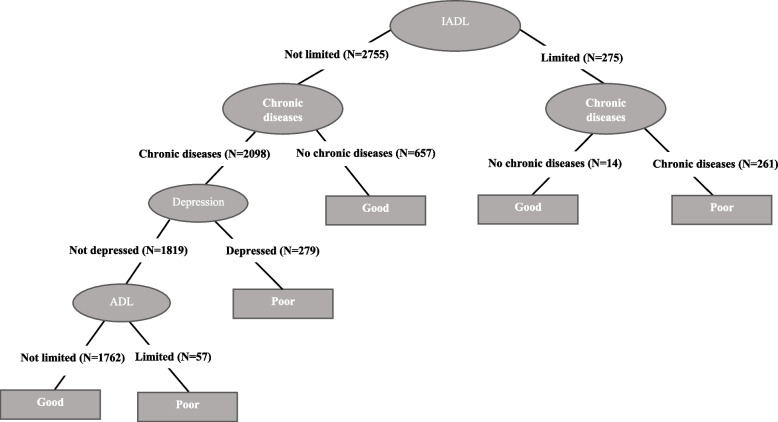
Decision tree for self-perceived health. High educated men

Figures 1, 2, 3, 4, 5 and 6 show the resulting decision trees for women and men, respectively, aged 65 to 79 by level of education. The most complex decision tree corresponds to women with a low level of education (Fig. 1), in which IADLs constitute the root node, that is, the variable with the greatest capacity to predict self-rated health. In this decision tree, with an accuracy of 72.2%, the right branch corresponds to women reporting IADL limitations whereas the left branch corresponds to those reporting no such limitations. This right branch comprises three additional nodes, defined, in order of importance as predictors of self-rated health, by ADL limitations, depression, and chronic diseases. These three nodes return a result of poor health in those cases in which an individual reports presenting one of these health conditions (i.e., an ADL limitation, depression or a chronic disease), whereas when an individual reports not presenting the corresponding health condition, a new node is opened culminating in the final node, represented by chronic diseases. In this right branch, only those that reach the end of the tree without reporting any IADL and ADL limitations, depression or chronic disease are classified as having good self-rated health. In contrast, the left branch, corresponding to those without any IADL limitations, comprises again three additional nodes defined by chronic diseases, depression, and ADL limitations. In this case, only those who report suffering a chronic disease and depression or an ADL limitation are predicted as having poor self-rated health.

In the case of women with a medium level of education (Fig. 2), the tree is less complex; in fact, this pattern becomes more generally established as we analyze women with a higher level of education. In women with medium education, where accuracy is 70.5%, ADL limitations constitute the root node, i.e., the variable with the greatest capacity to predict the real values of self-rated health. The left branch corresponds to those reporting no ADL limitations while the right branch represents those reporting at least one ADL limitation, resulting in poor self-rated health. In contrast, the left branch comprises three additional nodes, defined first by chronic diseases, followed by depression and, finally, IADL limitations. These three nodes provide a prediction of good health in those cases in which individuals report not suffering a chronic disease but also when they do but the condition is not accompanied by depression or any IADL limitations. All the other possibilities (i.e., being depressed or having an IADL limitation) provide a prediction of poor health.

Finally, in the case of highly educated women (Fig. 3), the decision tree is further simplified with only two nodes in addition to the root node, defined in this instance by IADL limitations. Although the tree is simpler, the level of accuracy remains high (73.6%). Here, the left branch corresponds to individuals that report presenting no IADL limitations while the right branch represents those reporting at least one of these limitations. The second node in the left branch is defined by ADL limitations, with a prediction of good health for those reporting no such limitations and of poor health for those suffering at least one ADL limitation. In the case of the right branch, the second node is defined by chronic diseases, with a prediction of good health for those who report not having any of these diseases and of poor health for those that do. It is interesting noting that for all educational groups, women who report both IADL and ADL limitations are always predicted to be in the poor self-rated health group. The combination “IADL: limited” and “ADL: limited” always appear in some branches to define poor self-rated health. Instead, other health conditions do not always lead to poor self-rated health.

In the case of the male respondents, there is almost no variation in the decision trees with level of education with the same pattern being observed for the prediction of self-rated health (Figs. 4, 5, and 6). The root node in each case is defined by IADL limitations and the tree presents a more complex left branch for those not reporting any such limitations that includes all the other health conditions. In these three trees, both the left and the right branches have a second node defined by chronic diseases; however, in the case of the right branch, this is the last node, with those reporting a chronic disease predicted to be in poor health and those reporting to be without a chronic disease predicted to be in good health. In the case of the left branch, the prediction ends in good health when an individual does not report any of these chronic diseases, whereas for those that do there are two more nodes defined by depression and ADL limitations. Among the male respondents, the fact of presenting depression is a prediction of poor health, whereas those that do not present depression continue along the classification tree to the last node defined by ADL limitations, with those presenting at least one such limitation predicted as being in poor health and those presenting no limitations predicted to be in good health.

The only exception to this general pattern is observed among low educated men (Fig. 4) for whom we observe an extra node in the right branch defined by ADL limitations when individuals report not having any chronic disease. In this case, the prediction ends in good health when they report no ADL limitations and in poor health when they do. This is the only case where the combination “IADL: limited” and “ADL: limited” appears in the trees and, as for women, leads to a prediction of poor self-rated health.

## Discussion

This study assesses the influence of various specific health conditions, both physical (IADLs, ADLs, chronic diseases) and mental (depression), in predicting responses about self-rated health in European women and men aged between 65 and 79 according to their educational attainment (low, medium, and high). The predictive capacity was assessed by means of classification trees based on the J48 algorithm, which belongs to the family of machine learning methods, and by using data from the sixth wave of the SHARE survey. The approach contributes to a better understanding of the health conditions that associate with individuals’ responses to the question about their general health, and how this relates with the health inequalities we observe when self-rated health is used as the health outcome.

The results presented here show the existence of differences in health conditions associated with the health perception of women and men. Indeed, in the case of women, these patterns are much more heterogeneous when the level of educational attainment is considered, whereas among men the pattern remains largely the same. The heterogeneous pattern described for female respondents does not, however, translate into different levels of accuracy as shown by the overlap between the confidence intervals of this indicator for the three educational groups. In fact, the simplest pattern, which is that of highly educated women, is the one that shows the highest levels of accuracy. However, we applied this same model to the cases of low and middle educated women as a sensitivity analysis but the results showed a significant reduction in the accuracy values (57% and 59% respectively for low and middle educated women) in the predictions of self-perceived health compared to the results obtained with the specific models. This result underscores the greater importance of education in identifying health inequalities among women compared to men. Among men, education does not lead to different associations among the four health conditions analyzed, though the higher the education, the higher the accuracy of the model. This seems to point, in the direction of the resource substitution theory, which states that the absence of one or more socio-economic resources may be offset by the greater influence of other resources [32]. In this instance, lower female participation in the labor market, as well as the higher gender wage gap, appears to have reinforced the importance of education for female health [33]. Here, the unequal health profile defined by education among women appears to have resulted in a different impact of each of the four health conditions when responding to the question regarding their overall health. The greater difference in prevalences observed among women would appear to explain the greater heterogeneity observed in the decision trees according to education among women, while the predictive pattern for men remains unchanged because of the smaller differences in health defined by education. This outcome serves to highlight the role that the social determinants of health appear to have on the process of the self-assessment of health, though with gender differences in their influence. In this case, education turns out to be an important factor in the process of normalization of women’s own health [10] as a factor that defines different patterns in the association of the four health determinants of self-perceived health, unlike in the case of men. Looking to the paths to poor self-perceived health beyond gender and educational differences, the combination of limitations, either ADL or IADL, and chronic diseases is shown to be always among the first two variables in all the different classification trees. It is noteworthy that those who report being limited in the case of ADLs always end up with a prediction of poor self-rated health, regardless of their gender and educational level. In the case of IADLs, despite almost always being the main node, they need to be combined with other health conditions such as chronic diseases or ADLs to end up with a prediction for the value of self-rated health. This highlights the fact that in the case of chronic diseases, what is significant in predicting self-rated health is precisely the absence of this type of disease, which leads to a prediction of good health. In the case of having a chronic disease, this fact is associated with other health conditions before ending up in a prediction of poor self-rated health. Finally, depression is always shown as the third node in the branches in which it appears, as a complement to chronic diseases among those people who do not report having any functional limitations. The mental dimension of health also appears combined with the IADL limitations in the case of women with a low educational level, again as the third node in this specific branch. This reinforces the idea of the complexity of the self-rated health indicator as a combination of different health outcomes with different paths that may bring to the same outcome. Moreover, this complexity is higher among subpopulations with a more disadvantaged health profile as low educated women. In other words, what seems to increase the complexity of the model is the increased presence of various health problems.

This study is not free of limitations. The fact of working with data from a survey implies that all responses are subject to the perception of the person interviewed. This implies that there is an effect of subjectivity in the response process itself to the questions for the four indicators used in this study to predict self-rated health. In addition, we have employed cross-sectional data; yet, there is a clear need in the future to analyze longitudinal data to test whether the effects of health conditions on self-rated health remain unchanged with age. A further limitation is the fact that the study analyzed aggregate data for Europe as a whole, without considering the specific characteristics of each country. It is hoped that this limitation can be addressed in future studies.

Finally, it is important to note that the analysis has been performed for the age range prior to the separation between the trend in mortality and self-rated health, which is also indicative of a change in self-reported health. In the future, it would be interesting to see if the results obtained are also applicable for ages 80 years and older. Therefore, conclusions from this research only apply to the age range between retirement and the reduction of the association between self-rated health and mortality.

## Conclusion

These results serve to highlight the weight that the step in the process prior to giving a response has on the final outcome, as stressed earlier by Jylhä [9]. It appears that the same variables that are being used to analyze health inequalities – which include education – also influence the individual process of evaluating one’s own health. This suggests that the same response to the self-rated health question may, in the case of women, represent very different health profiles in terms of the health conditions that define it. Self-rated health is a single health indicator that cannot be replaced by a single specific health indicator or a combination of these indicators. Its ability to summarize information from several dimensions of health at once offers unique characteristics to this health indicator.

The fact that the same factors that act as social determinants of health inequalities appear also to influence the decision-making process when reporting self-rated health has implications for the use and interpretation of this indicator. Future research needs to examine other possible social determinants of health – in addition to education – such as employment, because of their possible impact on the reference group used in defining self-rated health. Likely country differences must be also tested in line with the third factor proposed by Jylhä [9], cultural differences.

## Data Availability

The datasets generated and/or analysed during the current study are available in the web page of the SHARE Project, http://www.share-project.org/data-access.html.
